# Crop Pollination Exposes Honey Bees to Pesticides Which Alters Their Susceptibility to the Gut Pathogen *Nosema ceranae*

**DOI:** 10.1371/journal.pone.0070182

**Published:** 2013-07-24

**Authors:** Jeffery S. Pettis, Elinor M. Lichtenberg, Michael Andree, Jennie Stitzinger, Robyn Rose, Dennis vanEngelsdorp

**Affiliations:** 1 Bee Research Laboratory, USDA-ARS, Beltsville, Maryland, United States of America; 2 Department of Entomology, University of Maryland, College Park, College Park, Maryland, United States of America; 3 Cooperative Extension Butte County, University of California, Oroville, California, United States of America; 4 USDA-APHIS, Riverdale, Maryland, United States of America; Universidade de São Paulo, Faculdade de Filosofia Ciências e Letras de Ribeirão Preto, Brazil

## Abstract

Recent declines in honey bee populations and increasing demand for insect-pollinated crops raise concerns about pollinator shortages. Pesticide exposure and pathogens may interact to have strong negative effects on managed honey bee colonies. Such findings are of great concern given the large numbers and high levels of pesticides found in honey bee colonies. Thus it is crucial to determine how field-relevant combinations and loads of pesticides affect bee health. We collected pollen from bee hives in seven major crops to determine 1) what types of pesticides bees are exposed to when rented for pollination of various crops and 2) how field-relevant pesticide blends affect bees’ susceptibility to the gut parasite *Nosema ceranae*. Our samples represent pollen collected by foragers for use by the colony, and do not necessarily indicate foragers’ roles as pollinators. In blueberry, cranberry, cucumber, pumpkin and watermelon bees collected pollen almost exclusively from weeds and wildflowers during our sampling. Thus more attention must be paid to how honey bees are exposed to pesticides outside of the field in which they are placed. We detected 35 different pesticides in the sampled pollen, and found high fungicide loads. The insecticides esfenvalerate and phosmet were at a concentration higher than their median lethal dose in at least one pollen sample. While fungicides are typically seen as fairly safe for honey bees, we found an increased probability of *Nosema* infection in bees that consumed pollen with a higher fungicide load. Our results highlight a need for research on sub-lethal effects of fungicides and other chemicals that bees placed in an agricultural setting are exposed to.

## Introduction

Honey bees, *Apis mellifera*, are one of the most important pollinators of agricultural crops [1]. Recent declines in honey bee populations in many North American and European countries [2]–[4] and increasing cultivation of crops that require insects for pollination [5] raise concerns about pollinator shortages [5], [6]. Habitat destruction, pesticide use, pathogens and climate change are thought to have contributed to these losses [2], [7], [8]. Recent research suggests that honey bee diets, parasites, diseases and pesticides interact to have stronger negative effects on managed honey bee colonies [9], [10]. Nutritional limitation [11], [12] and exposure to sub-lethal doses of pesticides [13]–[16], in particular, may alter susceptibility to or severity of diverse bee parasites and pathogens.

Recent research is uncovering diverse sub-lethal effects of pesticides on bees. Insecticides and fungicides can alter insect and spider enzyme activity, development, oviposition behavior, offspring sex ratios, mobility, navigation and orientation, feeding behavior, learning and immune function [9], [13], [14], [16]–[22]. Reduced immune functioning is of particular interest because of recent disease-related declines of bees including honey bees [3], [23]. Pesticide and toxin exposure increases susceptibility to and mortality from diseases including the gut parasite *Nosema* spp. [14], [15]. These increases may be linked to insecticide-induced alterations to immune system pathways, which have been found for several insects, including honey bees [22], [24]–[26].

Surveys of colony food reserves and building materials (i.e. wax) have found high levels and diversity of chemicals in managed colonies [18], [27], [28]. These mixtures have strong potential to affect individual and colony immune functioning. However, almost all research to-date on pesticides’ effects on pathogen susceptibility fed a single chemical to test bees [16]. Because pesticides may have interactive effects on non-target organisms (e.g. [29]), it is crucial to determine how real world combinations and loads of pesticides affect bee health.

One pathogen of major concern to beekeepers is *Nosema* spp. The endoparasitic fungal infections of *N. apis* and *N. ceranae* adversely affect honey bee colony health, and can result in complete colony collapse [30]. Infection with *Nosema* in the autumn leads to poor overwintering and performance the following spring [31], and queens can be superseded soon after becoming infected with *Nosema* [32]. We chose *Nosema* as a model pathogen because earlier work [13], [14] had demonstrated an interaction with pesticide exposure.

This study addresses two important questions. 1) What types of pesticides might bees be exposed to in major crops? While multiple studies have characterized the pesticide profile of various materials inside a honey bee nest [27], [28], few have looked at the pollen being brought back to the nest. 2) How do field-relevant pesticides blends affect bees’ susceptibility to infection by the *Nosema* parasite?

## Methods

### Ethics Statement

Pollen was collected from honey bees with permission of the beekeepers and the land owners.

### Hive Selection and Pollen Collection

We collected pollen carried by foraging honey bees returning to the hive for nine hives in seven crops: almond, apple, blueberry, cranberry, cucumber, pumpkin, and watermelon (Table 1). For each crop, we selected three fields that were separated by at least 3.2 km. Hives were deployed in these fields for pollination services based on growers’ needs. Within each selected field, we chose the three honey bee hives with the strongest foraging forces by observing flight in the bee yard for 5–10 min, and attached plastic pollen traps (Brushy Mountain Bee Farm, Moravian Falls, NC) to these hives. Pollen traps collect the pollen pellets bees carry on their hind tibiae in flattened regions called corbiculae. Bees use this pollen to make food for larvae inside the nest. We checked traps after three days, and removed them if they contained at least 5 g of pollen. Traps with less than 5 g remained on hives until they contained 5 g of pollen or for 10 days. We placed pollen removed from traps in 50 mL centrifuge tubes and stored the samples on ice until they could be transferred to a −29°C freezer in the lab.

**Table 1:** Quantity and diversity of pollen collected in pollen traps on individual honey bee hives.

Crop	Location	Mean grams of pollen collected (se)	Mean number of pollen types (se)
Almond	Rosedale, CA; Kern County	42.0 (9.1)^a,b^	1.7 (0.2)^a,b^
Apple	York Springs, PA; Adams County	26.7 (2.6)^a^	4.9 (0.5)^c^
Blueberry	Deblois, ME; Washington County	4.1 (1.5)^b^	6.0 (1.0)^c^
Cranberry (early season)	Hammonton, NJ; Atlantic County	13.0 (2.5)^a,b^	4.0 (1.0)^b,c^
Cranberry (late season)	Hammonton, NJ; Atlantic County	13.9 (3.8)^a,b^	4.1 (0.6)^b,c^
Cucumber	Cedarville, NJ; Cumberland County	8.1 (2.7)^b^	5.5 (1.3)^b,c^
Watermelon	Seaford, DE; Sussex County	27.1 (11.2)^a,b^	7.1 (1.2)^c^
Pumpkin	Kutztown, PA; Berks County	98.6 (29.0)^a,b^	3.7 (0.6)^b,c^

Letters indicate statistically different groups.

Because our first round of pollen trapping in cranberry fields yielded little pollen, we collected pollen from each hive in cranberry fields twice: early in the flowering season and late in the season. We separate these samples in data analyses, referring to them as “Cranberry early” and “Cranberry late.”

We measured the wet weight of each pollen sample, and compared the quantity of pollen collected by hives in different crops via a Kruskal-Wallis test followed by a post-hoc non-parametric Tukey-type test (using the R package nparcomp [33]). We then divided each sample into three portions. A 5 g subsample was sorted by color and then each group of similarly colored pollen pellets were identified (see below); a 3 g subsample was sent to the USDA’s Agricultural Marketing Service Laboratory in Gastonia, NC for pesticide analysis; and a 10 g subsample was sent to the USDA-ARS Bee Research Laboratory (Beltsville, MD) for the *Nosema* infection study. Because almond pollen was collected after all other pollens, we were unable to include it in the pesticide analysis and *Nosema* infection study. In cases where the total amount of pollen collected from a single colony was less than 6 g all the pollen was used for pesticide analysis.

### Pollen Identification

Each 5 g pollen subsample was dehydrated in a drying oven at 40°C. We considered a sample to be dry when its weight did not change between two consecutive time points (measured every 4–6 h). Typically pollen dried in 12–18 h. To identify pollen types collected by the bees, we sorted the pollen in each subsample by color, quantified each color by comparing to Sherwin-Williams® color palettes, re-weighed after color separation and fixed each color from each subsample on a separate slide. We prepared each slide by grinding 2 pollen pellets in 2 mL water and letting them dissolve to form a slurry. We placed a small amount of slurry on a slide with a drop of silicon oil, and covered slides and sealed with clear nail polish after letting air bubbles escape for 48 h. We visually identified each pollen type under 400x magnification by comparing with published reference collections [34]–[36]. Visual identification of pollen grains through comparison with voucher or reference specimens is standard in pollination ecology [37], [38]. Similarities between closely related pollens, however, sometimes prevent identification to genus or species with this method [39]. Because of this limitation, we assumed that all pollen collected in apple (*Malus domestica*) orchards that was identified as *Malus* sp. was from apple trees, and that all pollen in the Cucurbitaceae family collected in cucumber (Cucurbitaceae, *Cucumis sativus*) fields was from cucumber flowers.

For each subsample, we estimated pollen diversity as the number of different pollen colors collected from that bee hive. We also calculated the proportion, by weight, of the pollen that was identified as belonging to the target crop’s genus. Many samples could only be identified to genus, so assessing target genus rather than target crop permitted a more inclusive analysis. We used Kruskal-Wallis tests to determine whether either of these measures differed with the crop in which sampled bee hives were placed.

### Pesticide Analysis

We determined the identity and load of pesticide residues present in pollen samples collected from all crops (except almond). For each field sampled (*n* = 19), we pooled pollen from the three hives for analysis. One early-season cranberry field and one cucumber field did not yield sufficient pollen in traps for pesticide analysis. Methods follow the LC/MS-MS and GC/MS methods for pollen analysis described in Mullin et al. [27]. We used these data to determine the total number of pesticides detected in each sample, each sample’s total pesticide load, and the diversity and load of pesticides in each of 10 categories: insecticides, fungicides, herbicides, and several insecticide types (carbamates, cyclodienes, formamidines, neonicotinoids, organophosphates, oxadiazines and pyrethroids). To permit comparison between categories with different numbers of elements, we calculated diversity as the proportion of pesticides from a category found in a given sample, and load as the total load divided by the number of chemicals in that category. We only calculated diversity for categories with at least three chemicals.

The total number of pesticides present and total load did not meet parametric assumptions. We thus analyzed how these variables differ between crops using non-parametric Kruskal-Wallis tests. When separated by category and log-transformed, pesticide loads did meet parametric assumptions. We thus determined whether load varied by pesticide category using a general linear mixed model with sample as a random effect, to control for the fact that our regression included one data point per category from each sample. Insufficient degrees of freedom prevented us from expanding this model to include crop. We thus asked whether the pesticide load and diversity varied with crop for each category using one Kruskal-Wallis test per category and applying a sequential Bonferroni correction [40] across pesticide categories to control for multiple comparisons.

### *Nosema* Infection

The *Nosema* infection experiment is similar to published methods [26]. We obtained 210 disease-free honey bees from each of three healthy colonies at the Bee Research Laboratory. Each bee was placed into one of 21 groups upon emergence, with the ten bees in the same group and from the same colony housed together in a wooden hoarding cage (12×12×12 cm). Each group of bees was fed 1 g of pollen mixed with 0.5 mL of syrup (1∶1 sucrose to water by weight), which they fully consumed in 2–4 days. These pollen cakes were placed in small petri dishes with the laboratory cages. Pollen from either one of the crop fields or one of two control diets were used. The pollen control group (“BRL”) was fed a mixed pollen diet prepared by the USDA-ARS Bee Research Laboratory. This pollen was collected in the desert Southwest (Arizona Bee Products, Tucson, AZ) and tested as pesticide-free by the USDA Agricultural Marketing Service prior to use. A protein control group was fed an artificial honey bee pollen substitute, MegaBee®. The *Nosema* inoculum was freshly prepared by mixing *Nosema* spores isolated from an infected colony (details provided in [26]) with 50% sucrose solution to obtain a concentration of ca. 2 million spores per 5 mL. We fed 5 mL of the *Nosema* inoculum to each cage during the first two days of adult life, then provided bees with *ad libitum* access to clean 50% (w/v) sucrose solution. We collected bees 12 days after infection and examined them for the presence or absence of *N. ceranae* spores by homogenizing individual abdomens in 1 mL distilled water. Here we focus only on infection prevalence, the number of individuals with *Nosema* spores.

To look for potential effects of individual pesticides on susceptibility to *Nosema* infection, we calculated the relative risk and its 95% confidence interval for bees becoming infected after consuming pollen with a specific pesticide. Relative risk measures the chance of developing a disease after a particular exposure [41], here each pesticide. A relative risk value of one indicates that the probability of infection is equal between exposed and non-exposed groups.

We further tested effects of pesticides in pollen on measured *Nosema* prevalence using a generalized linear mixed model with a bee’s *Nosema* status as the response variable, the source hive and pesticide variables as fixed effects, and the pollen sample fed to the bee as a random effect. Collinearity prevented developing a full model to investigate in detail how pesticides and pollen source affect bees’ susceptibility to *Nosema* infection. We thus selected for analysis two measures that vary with crop and are not nested: total pesticide diversity and fungal load. To graph logistic regression results in a meaningful manner, we followed recent recommendations [42], [43] and a modification of the logi.hist.plot function in the R popbio package [44] that shows our mixed model output.

## Results

### Pollen Collection

**Figure 1:**
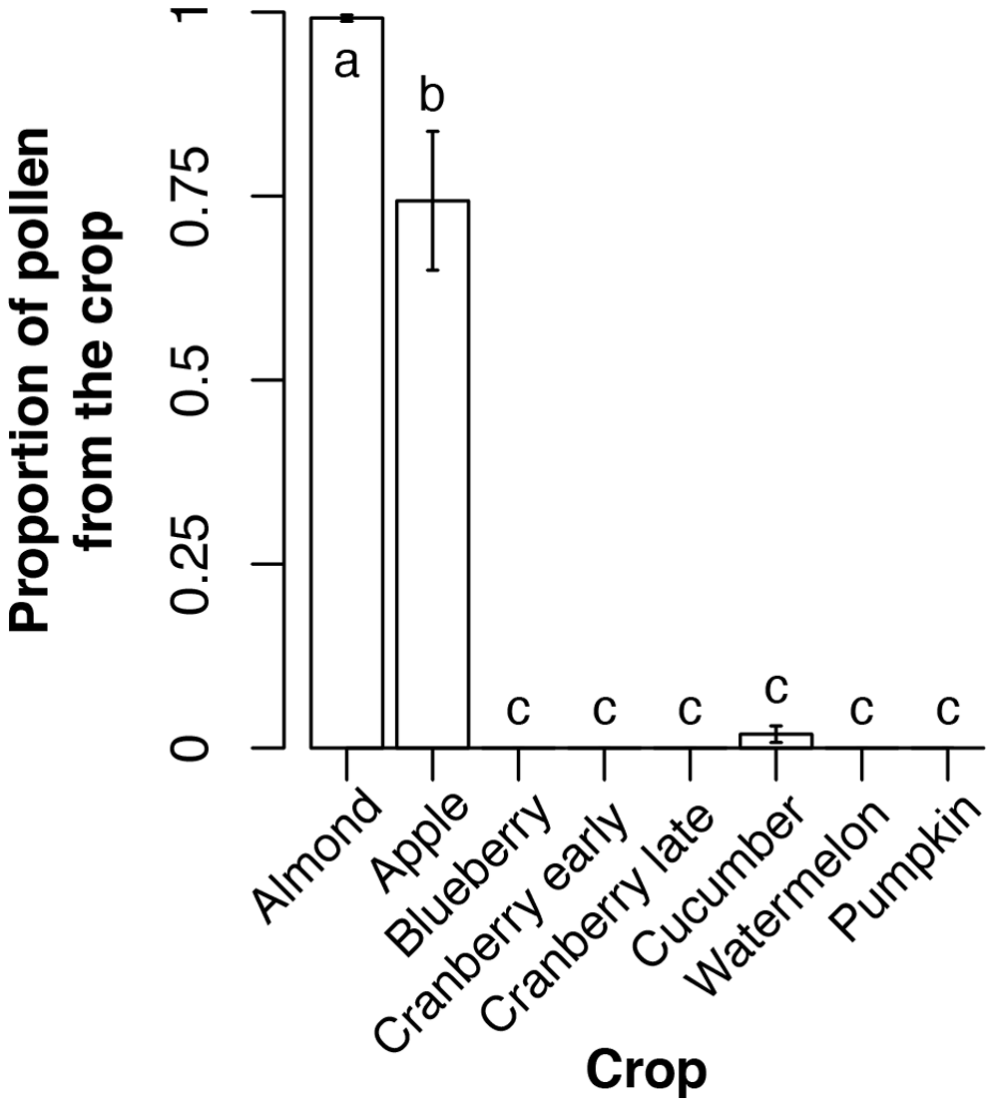
Pollen collection from the crop where a hive was located was low for most crops. Bars show mean ± se. Letters indicate statistically significant differences (*p*<0.05).

Bee colonies collected different amounts of pollen in the different crops (Table 1; Kruskal-Wallis test: H_7_ = 29.6, *p* = 0.0001). Pollen diversity, estimated by quantifying the number of differently colored pollen pellets collected in pollen traps, varied by crop (Table 1; Kruskal-Wallis test: H_7_ = 23.5, *p* = 0.0014). The proportion of pollen that bees collected from the target crop, except for almond and apple, was low (mean±se = 0.33±0.05; Table S1). Like pollen weights, this proportion dramatically differed between crops (Fig. 1; H_7_ = 44.86, *p*<0.0001). Notably, none of the pollen trapped from hives in blueberry, cranberry (early and late), pumpkin or watermelon fields was from the target crop.

### Pesticide Analysis

**Figure 2:**
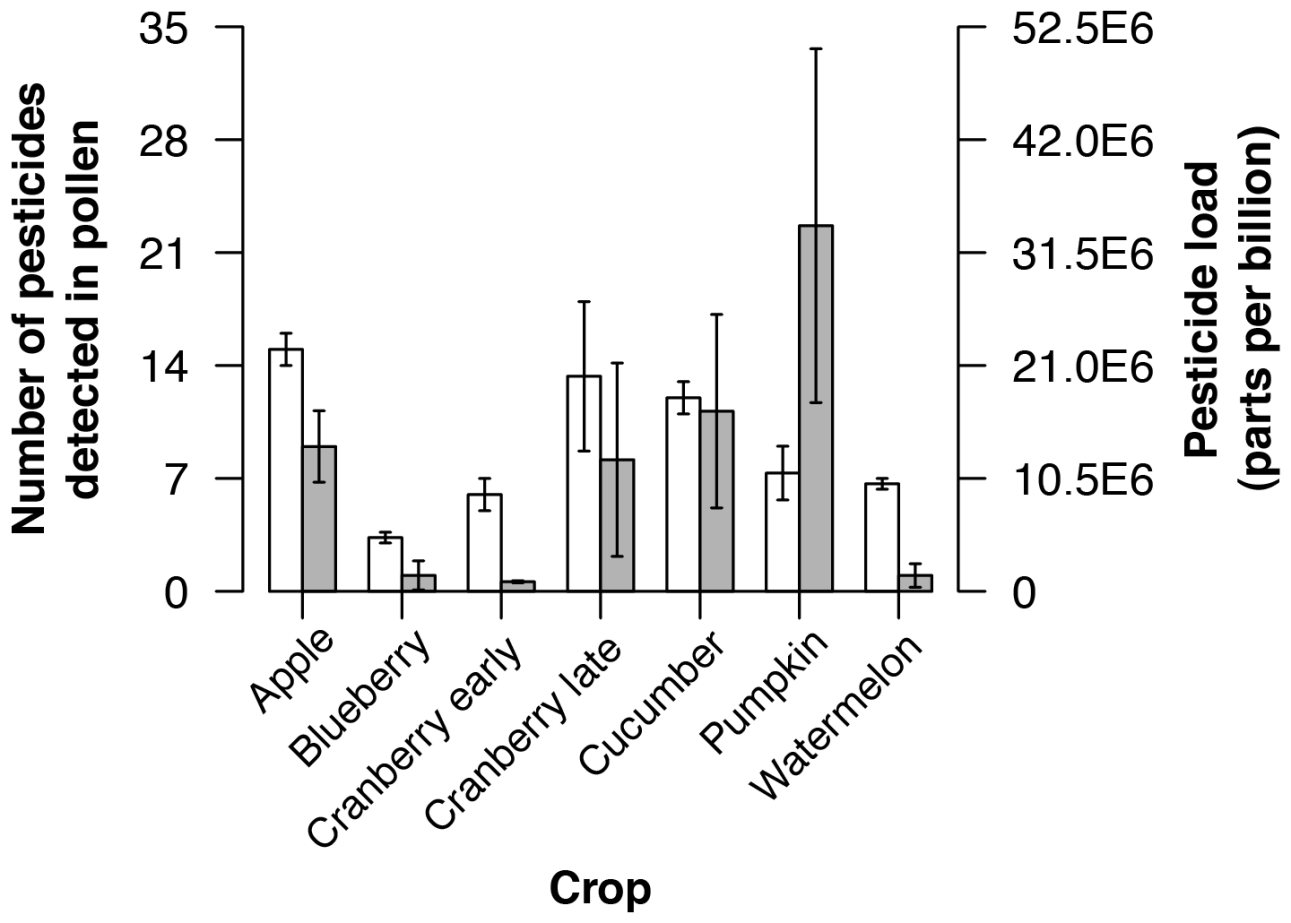
Pesticide diversity found in pollen samples, but not pesticide load, varied by crop. White bars show pesticide diversity, gray bars show pesticide load (mean ± se). Post-hoc testing found the following groups, where letters indicate statistically significant differences: apple a, b; blueberry c; cranberry_early d; cranberry_late b, d, e, f; cucumber e; pumpkin c, d, f; and watermelon d.

All pollen collected in this study contained pesticides (Table 2; mean ± se = 9.1±1.2 different chemicals, range 3–21). Pesticide loads ranged from 23.6 to 51,310.0 ppb (11,760.0±3,734.2 ppb). The maximum pesticide concentration in any single pollen sample exceeded the median lethal dose (LD_50_, the dose required to kill half a population within 24 or 48 h) for esfenvalerate and phosmet (Table 2). The number of pesticides detected in trapped pollen varied by the crop in which the bee hives were located (Kruskal-Wallis test: H_6_ = 12.96, *p* = 0.04), but the total pesticide load did not (H_6_ = 11.21, *p* = 0.08)(Fig. 2).

**Table 2:** Pesticides found in pollen trapped off honey bees returning to the nest.

Pesticide	Insecticide family	LD_50_ (ppm)^a^	Crops in which detected^c^	Detections	Quantity detected, mean±se (max) (ppb)	Relative risk (95% CI)
**Fungicides**						
Azoxystrobin		>1,562.5 [64]	Cr, Cu, Wa	10	60.3±25.6 (332)	0.75 (0.56, 1.02)
Captan		>78.13 [65]	Ap, Cr, Cu, Wa	9	976.9±734.4 (13,800)	0.59 (0.42, 0.81)†
Chlorothalonil		>1,414.06 [66]	Ap, Bl, Cr, Cu, Pu, Wa	17	4,491.2±2,130.7 (29,000)	2.31 (1.35, 3.94)†
Cyprodinil		>6,125 [67]	Ap	3	996.9±707.5 (12,700)	0.31 (0.15, 0.65)†
Difenoconazole		>781.25 [68]	Ap	3	171.4±119.4 (2,110)	0.31 (0.15, 0.65)†
Fenbuconazole		>2,282.65 [69]	Ap, Cr, Cu	10	227.3±89.2 (1,420)	0.33 (0.23, 0.48)†
Pyraclostrobin		573.44 [70]	Cr, Pu	4	2,787.1±1,890.1 (27,000)	2.85 (2.16, 3.75)†
Quintozene (PCNB)		>0.78 [71]	Cr	2	0.3±0.3 (4.7)	0.97 (0.59, 1.61)
THPI	Captan metabolite		Cr, Cu	3	832.1±531.8 (9,470)	0.42 (0.21, 0.82)†
**Herbicides**						
Carfentrazone ethyl		>217.97 [72]	Cr	1	0.1±0.08 (1.6)	1.05 (0.54, 2.05)
Pendimethalin		>388.28 [73]	Ap, Cr, Pu	5	5.1±3.7 (69.5)	1.47 (1.08, 1.99)†
**Insecticides**						
2,4 Dimethylphenyl formamide (DMPF)*	Amitraz (formamidine) metabolite		Bl, Cu, Pu, Wa	10	171.5±117.0 (2,060)	2.13 (1.56, 2.92)†
Acetamiprid	Neonicotinoid	55.47 [60]	Ap	3	59.1±32.2 (401)	0.31 (0.15, 0.65)†
Bifenthrin	Pyrethroid	0.11 [74]	Pu, Wa	3	6.6±3.8 (53.1)	2.08 (1.53, 2.83)†
Carbaryl	Carbamate	8.59 [75]	Ap, Cu, Wa	6	57.8±30.0 (403)	0.42 (0.27, 0.66)†
Chlorpyrifos	Organophosphate	0.86 [16]	Ap, Cr, Cu, Pu	7	3.1±1.1 (15.5)	0.89 (0.64, 1.23)
Coumaphos*	Organophosphate	35.94 [16]	Bl, Cr, Cu	6	2.2±1.0 (17.5)	0.62 (0.43, 0.91)†
Cyfluthrin	Pyrethroid	<0.31 [76]	Cr, Wa	2	0.6±0.4 (5.4)	1.31 (0.85, 2.02)
Cyhalothrin	Pyrethroid	0.30 [77]	Ap, Pu, Wa	7	14.6±7.9 (131)	0.94 (0.69, 1.29)
Cypermethrin	Pyrethroid	0.18–4.38 [78]	Cr	1	0.4±0.4 (6.9)	1.05 (0.54, 2.05)
Deltamethrin	Pyrethroid	0.39 [79]	Cr	1	4.5±4.5 (85.3)	1.05 (0.54, 2.04)
Diazinon	Organophosphate	1.72 [80]	Ap, Cr	3	1.4±1.0 (19.8)	0.56 (0.32, 0.97)†
Endosulfan I	Cyclodiene	54.69 [16]	Ap, Cr, Cu, Pu, Wa	8	1.5±0.7 (12.9)	1.60 (1.20, 2.14)†
Endosulfan II	Cyclodiene	54.69 [16]	Ap, Cr, Cu, Pu	6	0.8±0.3 (5.3)	1.41 (1.04, 1.91)†
Endosulfan sulfate	Endosulfan metabolite		Cr, Cu	4	0.3±0.2 (2.1)	0.79 (0.52, 1.19)
Esfenvalerate	Pyrethroid	0.13 [81]	Ap, Cr, Cu	7	16.9±12.0 (216)	0.51 (0.35, 0.75)†
Fluvalinate*	Pyrethroid	1.56 [82]	Bl, Cr, Cu, Pu, Wa	16	42.4±29.7 (570)	2.43 (1.49, 3.96)†
Heptachlor epoxide	Heptachlor^b^ (cyclodiene) metabolite		Cr	1	0.6±0.6 (12)	1.05 (0.54, 2.04)
Imidacloprid	Neonicotinoid	0.23 [83]	Ap	3	2.8±2.0 (36.5)	0.31 (0.15, 0.65)†
Indoxacarb	Oxadiazine	1.41 [84]	Ap	2	0.5±0.5 (9)	0.28 (0.11, 0.73)†
Methidathion	Organophosphate	1.85 [85]	Cr	1	1.6±1.6 (31)	1.05 (0.54, 2.04)
Methomyl	Carbamate	<3.91 [86]	Wa	1	13.6±13.6 (259)	1.54 (0.91, 2.61)
Phosmet	Organophosphate	8.83 [85]	Ap, Cr, Cu	5	798.7±772.4 (14,700)	0.36 (0.21, 0.61)†
Pyrethrins	Pyrethroid	0.16 [16]	Cr	1	5.1±5.1 (97.4)	1.05 (0.54, 2.05)
Thiacloprid	Neonicotinoid	114.06 [60]	Ap	2	1.1±0.8 (12.4)	0.35 (0.15, 0.82)†
**Control diets**						
BRL	NA	NA	NA	NA	NA	0.58 (0.23, 1.48)
MegaBee	NA	NA	NA	NA	NA	0.74 (0.33, 1.67)

a We divided LD_50_ values given as µg/bee (g) by 0.128 (equivalent to multiplying by 7.8) to obtain ppm when necessary [85]. If multiple values have been published, we include only the smallest.

b Heptachlor has been banned for use on cranberries since 1978 [87], but can persist in the soil for extended periods of time.

c Ap = apple, Bl = blueberry, Cr = cranberry, Cu = cucumber, Pu = pumpkin, Wa = watermelon.

* Used by beekeepers within the hive for parasitic mite control.

† Relative risk different from 1 at the 95% confidence level.

NA indicates information that is not relevant to control diets.

**Figure 3:**
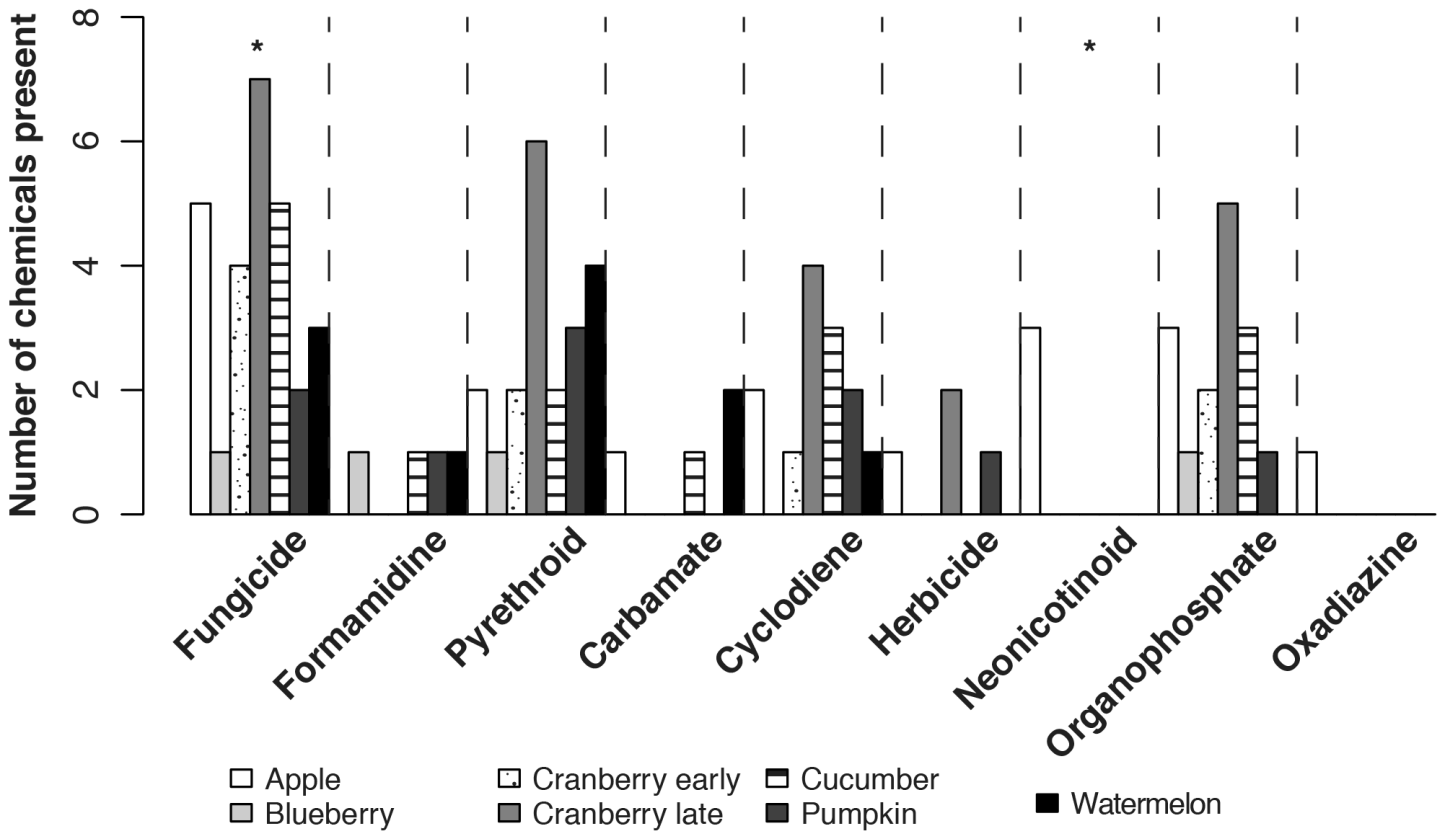
Fungicide and neonicotinoid diversities varied by crop. Bars show the total number of pesticides in each category found in each crop. Kruskal-Wallis test statistics comparing pesticide diversity between crops are: fungicides, H_6_ = 16.1, *p* = 0.01; cyclodienes, H_6_ = 6.9, *p* = 0.33; neonicotinoids, H_6_ = 17.9, *p* = 0.007; organophosphates, H_6_ = 14.3, *p* = 0.03; pyrethroids, H_6_ = 7.8, *p* = 0.26. We only compared pesticide diversities for categories containing at least three chemicals. Sequential Bonferroni adjusted critical values are: 0.01, 0.0125, 0.0167, 0.025, 0.05. A * indicates that the total number of pesticides varied between crops within that pesticide category.

**Figure 4:**
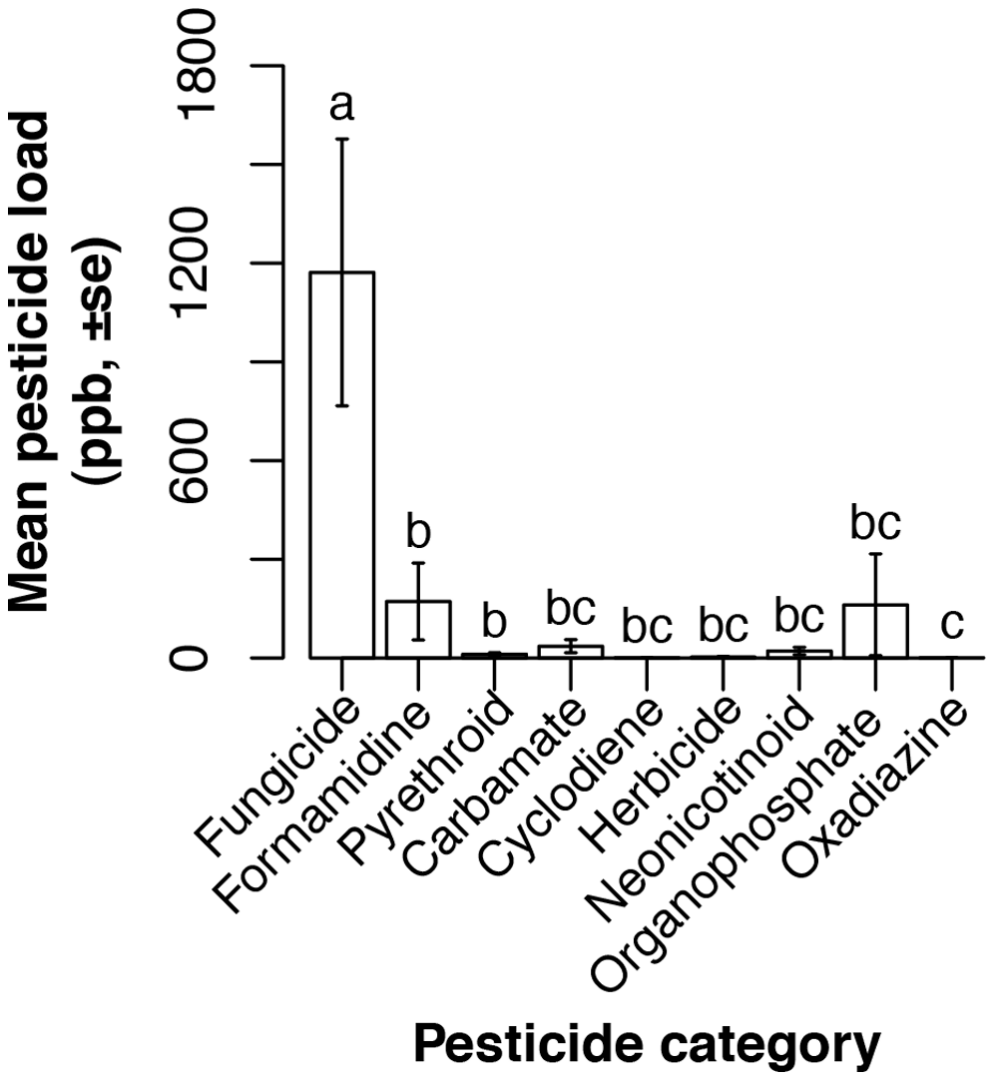
Load varied by pesticide category. Letters indicate statistically significant differences. The total load for each category is weighted by the number of chemicals in that category, to facilitate comparison across categories.

We found insecticides and fungicides in all 19, and herbicides in 23.6% of, pollen samples. Insecticides present in pollen collected by the bees came from seven categories. We found oxadiazines in 10.5%, neonicotinoids in 15.8%, carbamates in 31.6%, cyclodienes in 52.6%, formamidines in 52.6%, organophosphates in 63.2%, and pyrethroids in 100% of pollen samples. Both neonicotinoids and oxadiazines were present only in pollen collected by bees in apple orchards (Figs. 3, S1). Within a sample, pollen fungicide loads were significantly higher than loads of herbicides or any of the insecticide categories (Fig. 4; GLMM, likelihood ratio test: χ^2^ = 121.9, df = 8, *p*<0.0001).

After adjusting for multiple comparisons, pesticide loads did not vary by crop for any pesticide category (Fig. S1). We calculated pesticide diversity within only those categories containing three or more chemicals. Fungicide and neonicotinoid diversities varied by crop, but diversities of other pesticide categories did not (Fig. 3).

### *Nosema* Infection

147 of the 630 bees (23.3%) fed *Nosema* spores became infected. 22 of the 35 pesticides (62.9%) found in our pollen samples had relative risk values significantly different from 1 (Table 2). 8 pesticides (22.9%) were associated with increased *Nosema* prevalence, while the remaining 14 were associated with decreased *Nosema* prevalence. Two of the three detected pesticides applied by beekeepers to control hive mites (marked with a * in Table 2) had a relative risk larger than two, indicating *Nosema* prevalence in bees fed pollen containing those chemicals (DMPF and fluvalinate) was more than double the *Nosema* prevalence in bees that did not consume these chemicals. Of the seven pesticides found in pollen from over half, or at least four, of the crops, the majority were associated with higher *Nosema* prevalence in bees that consumed them. Both control diets had relative risk values not significantly different from one.

**Figure 5:**
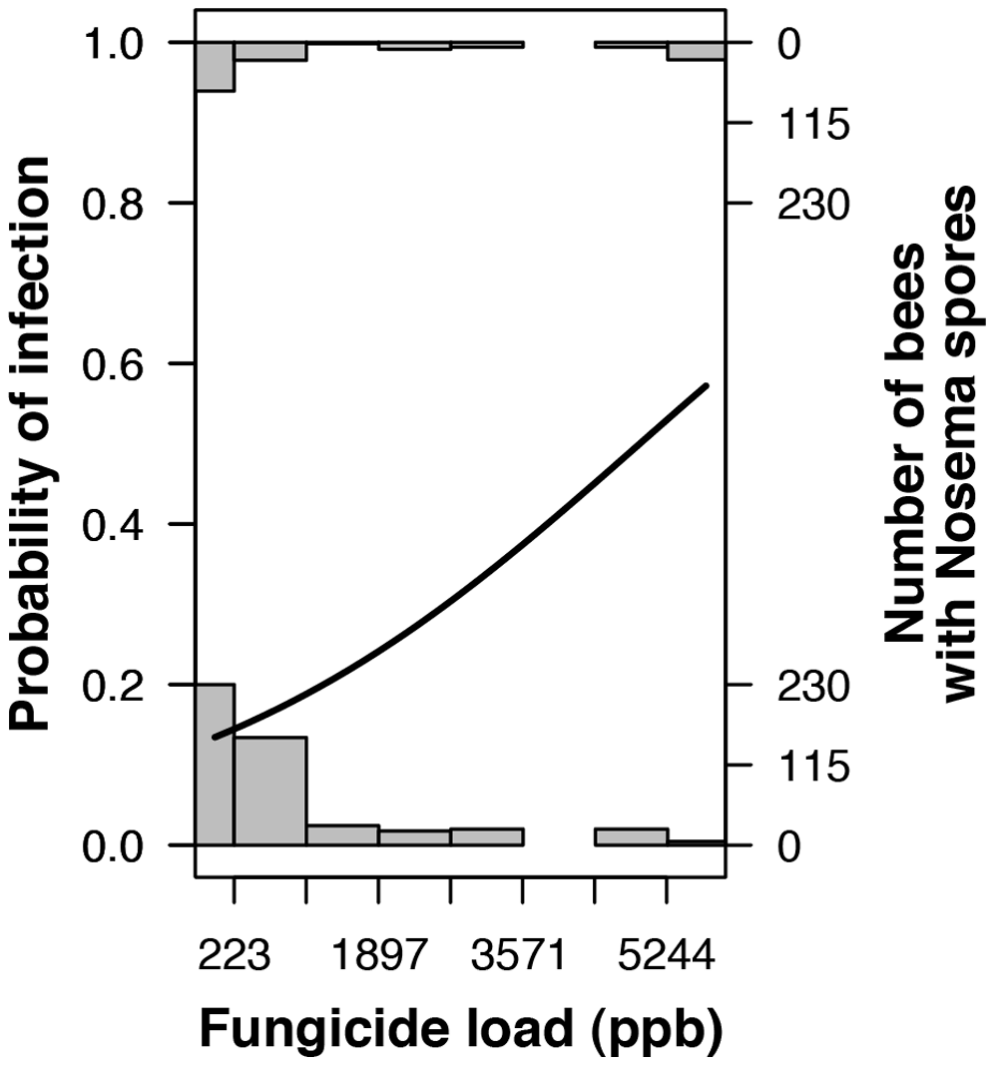
Probability of *Nosema* infection increased with fungicide load in consumed pollen. Histograms show the number of bees with (top) and without (bottom) *Nosema* spores as a function of the fungicide load in the pollen they were fed. The curve shows the predicted probability of *Nosema* infection.

A pollen sample’s fungicide load significantly affected *Nosema* prevalence among bees fed that pollen (Fig. 5; GLMM, likelihood ratio test: χ^2^ = 5.8, df = 1, *p* = 0.02), but pesticide diversity did not (χ^2^ = 1.7, df = 1, *p* = 0.19). A bee’s source colony, included as a blocking variable, also did not affect *Nosema* prevalence (χ^2^ = 2.0, df = 2, *p* = 0.36). Replacing fungicide load with chlorothalonil load obtained the same result (chlorothalonil load: χ^2^ = 5.3, df = 1, *p* = 0.02; pesticide diversity: χ^2^ = 1.5, df = 1, *p* = 0.23; source colony: χ^2^ = 2.0, df = 2, *p* = 0.36; fungicide load model AIC = 612.71, chlorothalonil load model AIC = 613.15). Chlorothalonil was also the most abundant fungicide in our samples, and comprised 50.0±10.2% (mean ± se) of the per sample total fungicide load.

## Discussion

The results from this study highlight several patterns that merit further attention. First, despite being rented to pollinate specific crops, honey bees did not always return to the nest with corbicular pollen from those crops. These findings support other research with honey bees and native bees indicating that in some crops native bees may be more efficient pollinators [45]. Second, fungicides were present at high levels in both crop and non-crop pollen collected by bees. Third, two fungicides (chlorothalonil and pyraclostrobin), and two miticides used by beekeepers to control varroa infestation (amitraz and fluvalinate) had a pronounced effect on bees’ ability to withstand parasite infection. Research on pesticides’ effects on bee health has focused almost exclusively on insecticides (e.g. fipronil [15] and the neonicotinoids imidacloprid [13], [14] and thiacloprid [15]). Finally, several individual pollen samples contained loads higher than the median lethal dose for a specific pesticide. While multiple studies have shown negative effects of specific pesticides on honey bee individual and colony health [14], [15], [22], [26] and high pesticide exposure [27], [28], ours is the first to demonstrate how real world pollen-pesticide blends affect honey bee health.

Our results show that beekeepers need to consider not only pesticide regimens of the fields in which they are placing their bees, but also spray programs near those fields that may contribute to pesticide drift onto weeds. The bees in our study collected pollen from diverse sources, often failing to collect any pollen from the target crop (Fig. 1). All of the non-target pollen that we were able to identify to genus or species was from wildflowers (Table S1), suggesting the honey bees were collecting significant amounts of pollen from weeds surrounding our focal fields. The two exceptions to this were hives placed in almond and apple orchards. Almond flowers early in the year, and almond orchards are large, thus providing honey bees with little access to other flowers. Honey bees rarely collect pollen from blueberry or cranberry flowers, which only release large quantities of pollen after being vibrated by visiting bees (buzz pollination) [46], [47]. Honey bees are not capable of buzz pollination and thus are unlikely to collect large amounts of pollen from these plants to bring back to the colony. Bumble bees, which can buzz pollinate, collect mainly blueberry pollen when placed in blueberry fields [48]. Interestingly, the two crops that saw high levels of pollen collection by honey bees are Old World crops that evolved with honey bees as natural pollinators. Crops native to the New World, where honey bees have been introduced, yielded little or no pollen in our samples.

It is possible that bees were exposed to pesticides while collecting nectar from our focal crops, even when we detected no pollen from those crops. Because pollen traps collect only corbicular pollen intended for consumption by the colony, our data indicate only flowers from which bees are actively collecting pollen and not all flowers they visited. Several studies have detected pesticides in floral nectar and pollen [49], [50], sometimes in concentrations with sublethal effects on honey and bumble bees [51], [52]. Honey bees may collect nectar from blueberry and cranberry flowers via legitimate visits or “robbing” through slits cut at the base of flower corollas [53]. However, exposure to pesticides via nectar may be unlikely in cucumber, pumpkin and watermelon. Beekeepers often report poor honey production when their hives are placed in these crops (pers. obs.).

The combination of high pesticide loads and increased *Nosema* infection rates in bees that consumed greater quantities of the fungicides chlorothalonil and pyraclostrobin suggest that some fungicides have stronger impacts on bee health than previously thought. *Nosema* infection was more than twice as likely (relative risk >2) in bees that consumed these fungicides than in bees that did not. Research on the sub-lethal effects of pesticides on honey bees has focused almost entirely on insecticides, especially neonicotinoids [54]. In our study, neonicotinoids entered the nest only via apple pollen. However, we found fungicides at high loads in our sampled crops. While fungicides are typically less lethal to bees than insecticides (see LD_50_ values in Table 2), these chemicals still have potential for lethal [55] and sub-lethal effects. Indeed, the fungicides chlorothalonil (found at high concentrations in our pollen samples) and myclobutanil increases gut cell mortality to the same degree as imidacloprid [56], an insecticide with numerous sub-lethal effects (e.g. [21], [57]). Exposure to fungicides can also make bees more sensitive to acaricides, reducing medial lethal doses [58]. In our study, consuming pollen with higher fungicide loads increased bees’ susceptibility to *Nosema* infection. This result is likely driven by chlorothalonil loads. The pesticide with the highest relative risk was the fungicide pyraclostrobin. Bees that consumed pollen containing pyraclostrobin were almost three times as likely (relative risk = 2.85, 95% CI 2.16–3.75; Table 2) than bees consuming pollen without this chemical to become infected after *Nosema* exposure. Our results show the necessity of testing for sub-lethal effects of pesticides on bees, and advocate for testing more broadly than the insecticides that are the targets of most current research.

A similarly large increased risk of *Nosema* infection was associated with consumption of DMPF and fluvalinate, miticides applied by beekeepers to help control the highly-destructive *Varroa* mite [3]. The path from in-hive application of these miticides to pollen on foragers returning to the hive is unclear. An increasingly popular practice, rotating combs out of hives to remove accumulated pesticides, is expected to reduce miticide levels in hives, and will hopefully decrease spread of these chemicals to the environment. Potential extra-nest sources, however, would slow efforts to reduce miticide accumulation and slow the development of resistance to these chemicals.

Insecticide relative risk values showed an interesting pattern: directional separation by insecticide family. Within a family, relative risk values significantly different than one were almost all in the same direction. The formamidine (DMPF) and two of the three the pyrethroids (bifenthrin and fluvalinate, but not esfenvalerate) were associated with an increased risk of *Nosema* infection. The carbamate (carbaryl), all neonicotinoids (acetamiprid, imidacloprid and thiacloprid), organophosphates (coumaphos, diazinon and phosmet) and the oxadiazine (indoxacarb) were associated with reduced risk of *Nosema* infection. Esfenvalerate and coumaphos have previously been found to be associated with apiaries without Colony Collapse Disorder [59]. These patterns suggest that insecticides’ modes of action have differential effects on honey bee immune functioning. Because of the relatively small number of pesticides we found in each insecticide family, however, additional sampling is necessary to determine how robust this pattern is.

The large numbers of pesticides found per sample and the high concentrations of some pesticides are concerning. First, two pollen samples contained one pesticide each at a concentration higher than the median lethal dose. Esfenvalerate (LD_50_ = 0.13 ppm) was measured at 0.216 ppm in pollen collected by bees in a cucumber field, and phosmet (LD_50_ = 8.83 ppm) at 14.7 ppm in one apple orchard. While the mean loads for these pesticides are well below their respective median lethal doses (0.0169 ppm for esfenvalerate, 0.7987 ppm for phosmet), our data indicate some bee colonies are being exposed to incredibly high levels of these chemicals. Second, research suggests that simultaneous exposure to multiple pesticides decreases lethal doses [58], [60] or increases supersedure (queen replacement) rate [61]. Our pollen samples contained an average of nine different pesticides, ranging as high as 21 pesticides in one cranberry field. Thus published LD_50_ values may not accurately indicate pesticide toxicity inside a hive containing large numbers of pesticides. Research looking at additive and synergistic effects between multiple pesticides is clearly needed. Third, pesticides can have sub-lethal effects on development, reproduction, learning and memory, and foraging behavior. The mean and maximum imidacloprid loads in our samples (0.0028 and 0.0365 ppm, respectively) are higher than some published imidacloprid concentrations with sub-lethal effects on honey and bumble bees (0.001–0.0098 ppm [21], [54], [62]).

It is not surprising that total pollen collection varied by crop. Bee foraging activity levels vary with weather [63], thus outcomes of short-term measurements may be sensitive to temperature, cloud cover or humidity during data collection. Because we collected pollen samples from different parts of the country and on different days, weather conditions undoubtedly differed between crops. Crop flowering timing and landscape-level floral availability can also affect bee activity levels. We focused our analyses on variables less affected by these factors, such as the diversity of pollen types found in samples and the proportion of a sample that was from the target crop.

Our results are consistent with previously published pesticide analyses of pollen collected by honey bees or honey bee nest material [16], [18], [27]. The more intensive and geographically more diverse sampling of Mullin et al. [27] resulted in almost triple the number of pesticides we found, but the average number of pesticides per sample (7.1) is slightly lower than our 9.1. In our study and those listed above, pesticides applied by beekeepers to control hive pests were present in a large proportion of the samples, often in quantities higher than most of the pesticides that are applied to crops.

Our results combined with several recent studies of specific pesticides’ effects on *Nosema* infection dynamics [13]–[15] indicate that a detrimental interaction occurs when honey bees are exposed to both pesticides and *Nosema*. Specific results vary, and may depend on the pesticide or dose used. For example, bees exposed to imidacloprid and *Nosema* can have lower spore counts than bees only infected with the pathogen but also exhibit hindered immune functioning [13]. Our study improves on previous methodologies by feeding pollen with real-world pesticide blends and levels that truly represents the types of exposure expected with pollination of agricultural crops. The significant increase in *Nosema* infection following exposure to the fungicides in pollen we found therefore indicates a pressing need for further research on lethal and sub-lethal effects of fungicides on bees. Given the diverse routes of exposure to pesticides we show, and increasing evidence that pesticide blends harm bees [16], , there is a pressing need for further research on the mechanisms underlying pesticide-pesticide and pesticide-disease synergistic effects on honey bee health.

## Supporting Information

Figure S1 **Pesticide loads did not differ by crop for any pesticide category.** Kruskal-Wallis test statistics comparing pesticide loads between crops are: fungicides, H_6_ = 10.6, *p* = 0.10; herbicides, H_6_ = 8.3, *p* = 0.22; carbamates, H_6_ = 13.4, *p* = 0.04; cyclodienes, H_6_ = 6.7, *p* = 0.35; formamidines, H_6_ = 13.6, *p* = 0.03; neonicotinoids, H_6_ = 17.8, *p* = 0.007; organophosphates, H_6_ = 14.5, *p* = 0.02; oxadiazines, H_6_ = 11.3, *p* = 0.08; pyrethroids, H_6_ = 9.6, *p* = 0.14. Sequential Bonferroni adjusted critical values are: 0.0055, 0.0063, 0.0071, 0.0083, 0.01, 0.0125, 0.0167, 0.025, 0.06.
(DOCX)

Table S1 Plant sources of pollens collected by bees placed in seven crops.
(DOCX)
