# Supplementary material for: Crop Pollination Exposes Honey Bees to Pesticides Which Alters Their Susceptibility to the Gut Pathogen Nosema ceranae
Source: PLoS One. 2013 Jul 24;8(7):e70182. doi: 10.1371/journal.pone.0070182 (PMC3722151; doi:10.1371/journal.pone.0070182)

**Crop pollination exposes honey bees to pesticides which alters their susceptibility to the gut pathogen *Nosema ceranae***

Jeffery S. Pettis, Elinor M. Lichtenberg, Michael Andree, Jennie Stitzinger, Robyn Rose and Dennis vanEngelsdorp

**Figure S1. Pesticide loads did not differ by crop for any pesticide category.**

Kruskal-Wallis test statistics comparing pesticide loads between crops are: fungicides, H_6_ = 10.6, *p* = 0.10; herbicides, H_6_ = 8.3, *p* = 0.22; carbamates, H_6_ = 13.4, *p* = 0.04; cyclodienes, H_6_ = 6.7, *p* = 0.35; formamidines, H_6_ = 13.6, *p* = 0.03; neonicotinoids, H_6_ = 17.8, *p* = 0.007; organophosphates, H_6_ = 14.5, *p* = 0.02; oxadiazines, H_6_ = 11.3, *p* = 0.08; pyrethroids, H_6_ = 9.6, *p* = 0.14. Sequential Bonferroni adjusted critical values are: 0.0055, 0.0063, 0.0071, 0.0083, 0.01, 0.0125, 0.0167, 0.025, 0.06.


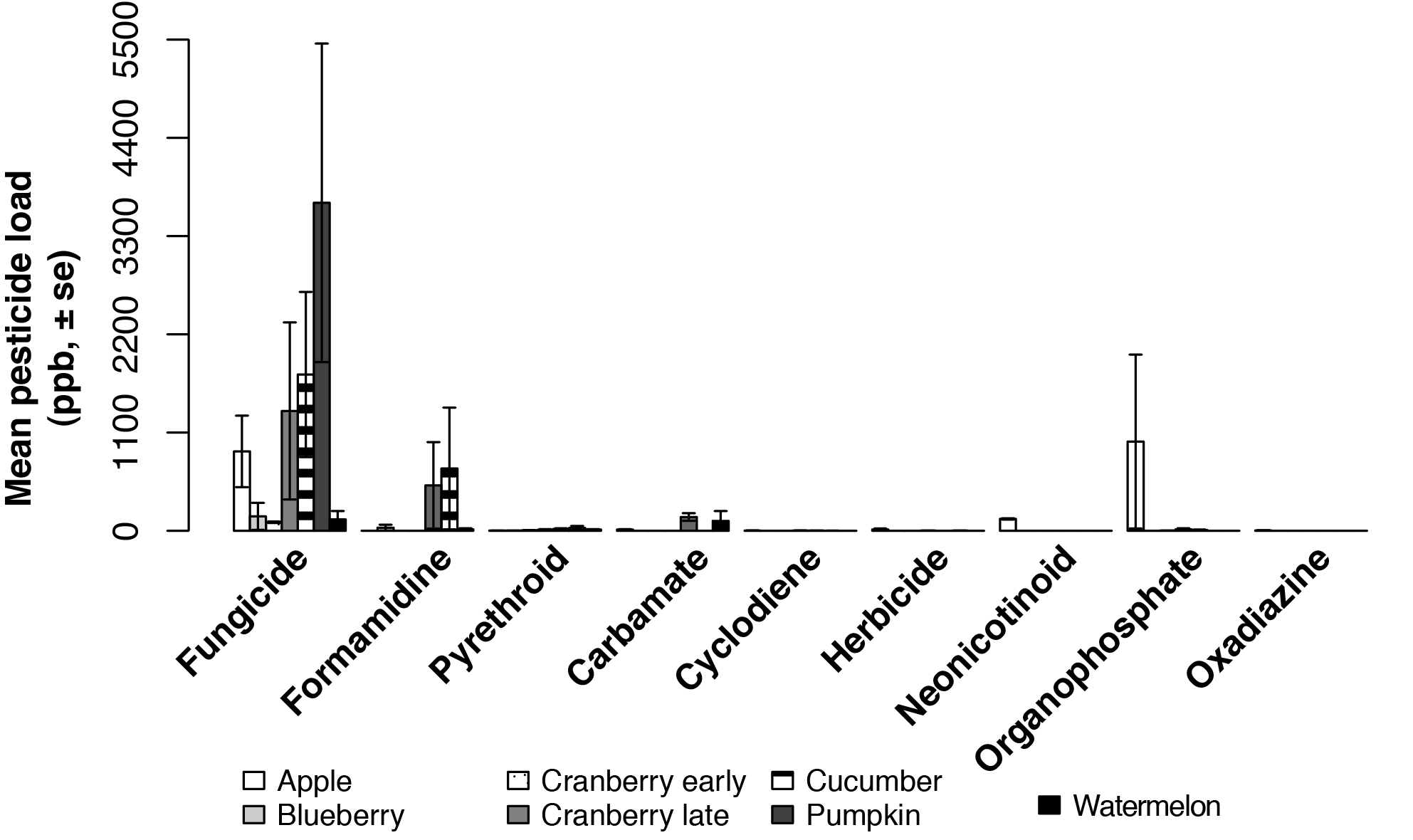

Supplement: Figure S1 — Pesticide loads did not differ by crop for any pesticide category. Kruskal-Wallis test statistics comparing pesticide loads between crops are: fungicides, H6 = 10.6, p = 0.10; herbicides, H6 = 8.3, p = 0.22; carbamates, H6 = 13.4, p = 0.04; cyclodienes, H6 = 6.7, p = 0.35; formamidines, H6 = 13.6, p = 0.03; neonicotinoids, H6 = 17.8, p = 0.007; organophosphates, H6 = 14.5, p = 0.02; oxadiazines, H6 = 11.3, p = 0.08; pyrethroids, H6 = 9.6, p = 0.14. Sequential Bonferroni adjusted critical values are: 0.0055, 0.0063, 0.0071, 0.0083, 0.01, 0.0125, 0.0167, 0.025, 0.06. (DOCX) [file pone.0070182.s001.docx]
