# Supplementary material for: Crop Pollination Exposes Honey Bees to Pesticides Which Alters Their Susceptibility to the Gut Pathogen Nosema ceranae
Source: PLoS One. 2013 Jul 24;8(7):e70182. doi: 10.1371/journal.pone.0070182 (PMC3722151; doi:10.1371/journal.pone.0070182)
Supplement: Table S1 — Plant sources of pollens collected by bees placed in seven crops. (DOCX) [file pone.0070182.s002.docx]

**Crop pollination exposes honey bees to pesticides which alters their susceptibility to the gut pathogen *Nosema ceranae***

Jeffery S. Pettis, Elinor M. Lichtenberg, Michael Andree, Jennie Stitzinger, Robyn Rose and Dennis vanEngelsdorp

**Table S1. Plant sources of pollens collected by bees placed in seven crops.**

| **Crop** | **Plant** | **% of pollen by weight, across all hives within a crop** |
| --- | --- | --- |
| Almond | Rosaceae, *Prunus dulcis* (almond) | 99.2 |
|  | Unknown | 0.8 |
| Apple | Rosaceae, *Malus* sp. (apple) | 74.2 |
|  | Asteraceae, *Taraxacum officinale* (common dandelion) | 6.4 |
|  | Brassicaceae, *Brassica* sp. (mustards) | 0.2 |
|  | Geraniaceae, *Geranium bicknellii* (Bicknell’s cranesbill) | 0.2 |
|  | Salicaceae, *Salix* spp. (willows) | 18.4 |
|  | Unknown | 0.6 |
| Blueberry | Ericaceae, *Vaccinium* *angustifolium* (low-bush blueberry) | 0.0 |
|  | Asteraceae, *Taraxacum officinale* (common dandelion) | 0.9 |
|  | Caprifoliaceae, *Diervilla lonicera* (northern bush honeysuckle) | 0.8 |
|  | Caprifoliaceae, *Viburnum* spp. (viburnums) | 57.1 |
|  | Cyperaceae, *Carex* spp. (sedges) | 11.4 |
|  | Pinaceae, *Pinus strobus* (eastern white pine) | 11.3 |
|  | Pinaceae, *Pinus* sp. (pine) | 0.4 |
|  | Rosaceae, *Rosa multiflora* (multiflora rose) | 18.2 |
| Cranberry_early | Ericaceae, *Vaccinium* *oxycoccos* (common cranberry) | 0.0 |
|  | Brassicaceae, *Rorippa palustris* (bog yellowcress) | 6.2 |
|  | Fabaceae spp. | 2.5 |
|  | Iridaceae, *Iris* spp. (irises) | 68.7 |
|  | Iridaceae sp. | 16.2 |
|  | Nymphaeaceae, *Nymphaea odorata* (American waterlily) | 5.3 |
|  | Oleaceae, *Ligustrum* sp. (privets) | 1.1 |
|  | Unknown | 0.09 |
| Cranberry_late | Ericaceae, *Vaccinium* *oxycoccos* (common cranberry) | 0.0 |
|  | Asteraceae, *Cichorium intybus* (common chicory) | 0.1 |
|  | Asteraceae, *Taraxacum officinale* (common dandelion) | 3.7 |
|  | Fabaceae spp. | 35.0 |
|  | Iridaceae, *Iris* spp. (irises) | 20.8 |
|  | Iridaceae, *Sisyrinchium* sp. (blue-eyed grasses) | data missing |
|  | Nymphaeaceae, *Nymphaea odorata* (American waterlily) | 11.8 |
|  | Oleaceae, *Ligustrum* sp. (privets) | 0.004 |
|  | Rosaceae sp. | 8.2 |
|  | Vitaceae spp. | 17.0 |
|  | Unknown | 3.4 |
| Cucumber | Cucurbitaceae, *Cucumis sativus* (cucumber) | 1.1 |
|  | Asteraceae, *Centaurea* sp. (knapweeds) | 1.9 |
|  | Asteraceae sp. | 1.0 |
|  | Cucurbitaceae sp. | 0.9 |
|  | Fabaceae, *Lathyrus* sp. (peas) | 3.6 |
|  | Fabaceae, *Melilotus* sp. (sweet clovers) | 1.0 |
|  | Fabaceae, *Vicia* sp. (vetches) | 25.2 |
|  | Fabaceae sp. | 63.2 |
|  | Unknown | 2.0 |
| Pumpkin | Cucurbitaceae, *Cucurbita pepo* (Jack-o-lantern pumpkin) | 0.0 |
|  | Asteraceae, *Centaurea* sp. (knapweeds) | 0.1 |
|  | Asteraceae spp. | 37.5 |
|  | Balsaminaceae, *Impatiens* sp. (touch-me-nots) | 1.3 |
|  | Chenopodiaceae spp. | 8.7 |
|  | Cruciferaceae, *Brassica* sp. (mustards) | 0.2 |
|  | Fabaceae, *Phaseolus* spp. (beans) | 5.2 |
|  | Fabaceae sp. | 34.1 |
|  | Lamiaceae sp. | 0.9 |
|  | Unknown | 11.9 |
| Watermelon | Cucurbitaceae, *Citrullus lanatus* (watermelon) | 0.0 |
|  | Asteraceae, *Helianthus* sp. (sunflowers) | 0.3 |
|  | Asteraceae, *Solidago* sp. (goldenrods) | 0.2 |
|  | Asteraceae, *Taraxacum officinale* (common dandelion) | 21.0 |
|  | Asteraceae sp. | 0.3 |
|  | Betulaceae, *Alnus* sp. (alders) | 2.1 |
|  | Betulaceae, *Betula* sp. (birches) | 0.07 |
|  | Betulaceae sp. | 11.6 |
|  | Chenopodiaceae, *Salsola* sp. (Russian thistles) | 0.2 |
|  | Fabaceae, *Phaseolus* sp. (beans) | 2.8 |
|  | Fabaceae spp. | 19.6 |
|  | Lamiaceae sp. | 0.05 |
|  | Unknown | 41.8 |
